# Application of HFCT and UHF Sensors in On-Line Partial Discharge Measurements for Insulation Diagnosis of High Voltage Equipment

**DOI:** 10.3390/s150407360

**Published:** 2015-03-25

**Authors:** Fernando Álvarez, Fernando Garnacho, Javier Ortego, Miguel Ángel Sánchez-Urán

**Affiliations:** 1 Department of Electrical Engineering, Polytechnic University of Madrid, Ronda de Valencia 3, Madrid 28012, Spain; E-Mail: miguelangel.sanchezuran@upm.es; 2 LCOE-FFII, Eric Kandel 1, Getafe 28906, Spain; E-Mail: fernandog@lcoe.etsii.upm.es; 3 DIAEL, Peñuelas 38, Madrid 28005, Spain; E-Mail: javier.ortego@diael.com

**Keywords:** partial discharges, HFCT sensors, UHF sensors, wideband PD measurements, insulation condition, on-line PD measurements, noise rejection, pattern recognition

## Abstract

Partial discharge (PD) measurements provide valuable information for assessing the condition of high voltage (HV) insulation systems, contributing to their quality assurance. Different PD measuring techniques have been developed in the last years specially designed to perform on-line measurements. Non-conventional PD methods operating in high frequency bands are usually used when this type of tests are carried out. In PD measurements the signal acquisition, the subsequent signal processing and the capability to obtain an accurate diagnosis are conditioned by the selection of a suitable detection technique and by the implementation of effective signal processing tools. This paper proposes an optimized electromagnetic detection method based on the combined use of wideband PD sensors for measurements performed in the HF and UHF frequency ranges, together with the implementation of powerful processing tools. The effectiveness of the measuring techniques proposed is demonstrated through an example, where several PD sources are measured simultaneously in a HV installation consisting of a cable system connected by a plug-in terminal to a gas insulated substation (GIS) compartment.

## 1. Introduction

The analysis of the types of defects and the degradation modes in different insulation materials of HV electrical systems has shown that the presence of PD is a very common characteristic in all of them [1,2,3]. Once PD activity is detected the identification and location of the associated type of defect is very important to evaluate whether the discharges are harmful or not.

On-line PD measurements have become a common practice for assessing the insulation condition of installed HV equipment. This type of testing is carried out during normal operation of the electrical system. For utilities, the most interesting advantage of on-line PD measurements is that once the sensors are installed in the power grid, the electrical service is not interrupted for the measurements. Another advantage of on-line tests is that PD activity can be acquired under different load conditions in temporal or permanent monitoring applications, which results very useful for the identification of certain types of defects and allows the analysis of the defects’ evolution over time.

Among the different PD measuring techniques available (electrical, acoustic, optical and analysis of chemical byproducts) [4,5,6,7,8], the electrical method is the most widely used because of its effectiveness. Despite the usefulness of this technique, the presence of electric noise disturbances when on-line PD tests are performed represents a drawback due to the loss of sensitivity, especially when PD pulses with low energy are present in the HV installation. Another drawback of on-line PD measurements appears when more than one source of pulse-shaped signals is present in the electric HV system under testing. In these cases, an adequate selection of the non-conventional measuring technique with the most suitable sensors and the implementation of effective signal processing tools are essential to achieve a correct evaluation of the insulation condition.

Different approaches have been developed and applied to PD measurement and processing techniques in order to achieve accurate diagnoses. Most of them are focused on wideband measurements with different types of sensors [5,9,10], noise rejection techniques [11], pulse classification processes [12,13,14] and defect location and identification [15,16]. In the study presented herein a complete measuring and processing method providing a comprehensive solution for the most common problems arising in on-line measurements is described.

The advantages of the complementary measurements applying non-conventional methods measuring with high frequency current transformers (HFCT) and UHF sensors are analyzed. These measurements performed with a developed measuring device integrated with powerful processing tools enable to perform better diagnosis and thus to improve the electrical system reliability.

In Section 2 the technical details and practical implementations of HFCT and UHF sensors for their application in complementary PD measurements are analysed and the sensors used in this study are described. In this section a UHF-HF converter designed to measure pulses captured in the UHF range using PD instruments for the acquisition of signals in the HF range is also described. In the following section the measuring instrument developed is described, together with three signal processing tools specially designed for on-line PD tests. Finally, experimental PD measurements applying HF and UHF sensors are performed according with a proposed measuring procedure and the results obtained are analyzed in detail.

## 2. PD Sensors and Frequency Converter for PD Measurements in UHF

In the detection of PD pulses by electromagnetic methods, two techniques are mainly distinguished, those that apply the conventional method based in the standard IEC 60270, in which PD pulses are measured in a frequency range below 1 MHz and those that implement non-conventional methods based in the use of sensors measuring in the HF (3–30 MHz), VHF (30–300 MHz) and UHF (300 MHz–3 GHz) frequency ranges [4,17,18]. A new technical specification (IEC 62478) which also covers non-conventional electrical methods in addition to the acoustical technique is currently under consideration. It is expected that this technical specification will be published in 2015.

The conventional method is used as reference for the quality assurance of the elements of HV electrical systems. This method, while suitable for laboratory measurements, is not appropriate for on-line measurements, since the noise conditions in most cases are very high for the measuring frequencies specified in the standard (≤1 MHz). To achieve an appropriate sensitivity in on-line measurements and to analyze in detail the captured signals, non-conventional methods with larger frequency ranges and bandwidths than those used according to the IEC 60270 standard are applied.

Moreover, to perform the measurements applying the conventional method, a coupling capacitor in series with a measuring impedance are used. In non-conventional methods the PD signals are captured with the sensors coupled directly in the HV equipment and the external coupling capacitor is not required, which suppose another advantage for on-line tests.

During the last three decades, the use of non-conventional methods implemented with HF and UHF sensors for on-line PD measurements has been generalized in order to detect incipient insulation defects. Theoretical and practical aspects to be known about these sensors are considered in this section.

Furthermore, different measuring devices have been designed to perform non-conventional PD tests. A measuring instrument has been developed by the authors together with a UHF-HF converter to measure the signals captured with both UHF and HF sensors. The frequency converter connected to the output of UHF sensors allows processing the signals acquired with less costly acquisition cards used to measure in the HF range. The UHF-HF converter developed and the advantages of its implementation are described in the last point of this section.

### 2.1. HFCT Sensors

**Figure 1:**
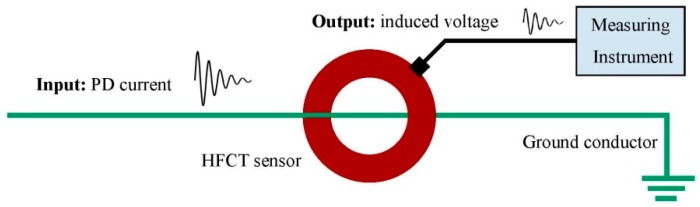
HFCT sensor placed in a ground conductor for PD measurement.

HFCT sensors are widely used for PD detection and their application for the location and identification of PD sources is very effective. A HFCT sensor also called radio frequency current transducer (RFCT) consists of an induction coil with a ferromagnetic core suitable for the measurement of transient signals as PD or pulse-shaped noise interferences. In general, when on-line PD measurements are performed on HV installations, HFCT sensors are clamped in the grounding conductors of the earthing network. For this application, the sensor can be modeled as a system in which the input is the current of the PD pulses flowing through it and the output is the induced voltage that is measured over the input impedance of the measuring instrument (usually 50 Ω), see Figure 1.

The transfer function of these magnetic sensors V = f(B) can be expressed by the Faraday’s law of induction.

(1) $$e=-n\cdot \frac{d\text{Φ}}{dt}=-n\cdot A\cdot \frac{dB}{dt}=-\mu_{0}\cdot n\cdot A\cdot \frac{dH}{dt}$$

where Φ is the magnetic flux passing through the coil of the secondary side which is formed by a number of turns n and presents an area A. In the case of a coil with a ferromagnetic core, Equation (1) can be written as Equation (2):

(2) $$e=-\mu_{0}\cdot \mu_{r}\cdot n\cdot A\cdot \frac{dH}{dt}$$

The induced voltage in the secondary is proportional to the rate of change of current in the primary, being the mutual inductance between the earth conductor and the secondary M, the proportional constant.

(3) $$e=M\cdot \frac{di}{dt}$$

Air-coil sensors present linearity for large frequency bandwidths; however the sensitivity of the sensor improves significantly when soft magnetic materials are used for the transformer core. The ferromagnetic core introduces to the transfer function of the sensor some nonlinear factors which depend mainly on the frequency but also on the temperature and flux density. For this reason this type of sensors should be designed according with a specified frequency characteristic [19].

In the origin of the discharge the current pulses generated have rise times of a few units of nanosecond or even below 1 nanosecond, presenting a frequency spectrum with significant components up to hundreds of MHz or even units of GHz [18,20,21,22]. The high frequency components of the PD signals are significantly filtered when the pulse propagates to the earth conductors where the HFCT sensors are placed. For the detection and location of PD events in electrical grids only the frequency components below 10 MHz are of interest [23].

**Figure 2:**
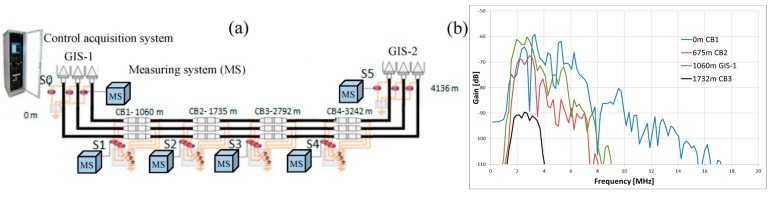
(**a**) On-line PD measurement with a monitoring application in a 45 kV XLPE power cable system; (**b**) Frequency spectrum of a PD pulse measured in different positions.

Figure 2b shows the frequency spectrum of a PD pulse measured in different positions along a power cable system using an on-line monitoring system. The pulse was generated in an insulation defect of a cable joint in the position CB1 and was captured by the HFCT sensors located in the positions CB1 (0 m), CB2 (675 m), CB3 (1732) and GIS-1(1060), see Figure 2a. The bandwidth of the sensors used is 100 kHz–25 MHz and the sampling rate of the acquisition board is 100 MS/s. It was observed an attenuation for the upper frequencies with the increasing distance, due to the behavior of the propagation path as a low-pass filter for PD pulses. Furthermore, the frequency content of the recorded pulses was always below 20 MHz. Considering this analysis and in order to comply with appropriate requirements of sensitivity adopting a good economical solution, the use of HFCT sensors with a bandwidth from hundreds of kHz to 20 MHz is recommended for their practical implementation in PD measurements.

PD measurements in the HF range using HFCT sensors provide considerable advantages:

- The sensitivity is not so pulse shape dependent as in conventional PD measuring instruments.
- The signal to noise ratio (SNR) can be improved, analyzing the data in certain frequency bands.
- High sensitivity is obtained when the sensors are located closed to the PD source and also when they are far from it. In a power cable system, when a PD pulse propagates through the cable shield, although the high frequency content of the signal is filtered, the pulse can be measured at distances exceeding one kilometer holding their spectral content up to units of megahertz [23].
- If two or more HFCT sensors are placed in a HV installation, the measurement of the PD pulses with a common time reference allows the determination of the location of defects by the time-of-flight analysis.
- PD pulses waveform can be recorded for post processing purposes. The recorded signals can be classified by the characterization of the pulse shape with the aim of discriminate different PD or noise sources. A proper classification of the recorded pulses and a subsequent analysis of the associated phase resolved PD (PRPD) patterns, improves the sensibility in the detection of defects and facilitates more accurate diagnoses.
- For the frequency range specified, ferrite cores are commonly available and a high quality HFCT sensor manufacture results easy and inexpensive.

**Figure 3:**
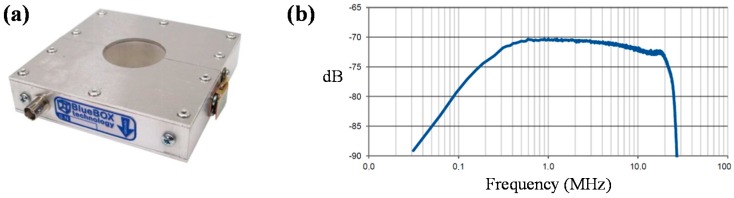
(**a**) Clamp type HFCT sensor; (**b**) Frequency response.

In this study, a clamp-type HFCT sensor has been designed for the measurement of PD in the ground connections of HV equipment in electrical power installations, see Figure 3a. Figure 3b shows the frequency response of the sensor, which presents a bandwidth from 100 kHz to 20 MHz.

### 2.2. UHF Sensors

Although UHF sensors for PD measurements are considered antennas, they do not measure in the far-field but rather in the near-field, where the power of the electromagnetic waves of PD signals is sufficiently significant. UHF sensors can be classified as internal or external couplers, according to whether they are mounted inside or outside the HV equipment. Internal UHF sensors are normally assembled during construction in the enclosure of GIS compartments, occasionally in power transformers and they can also be embedded in cable accessories. External UHF sensors consist of portable couplers that are fitted in inspection windows or in exposed barrier edges in GIS, transformers and other HV elements such as metal enclosed switchgear or rotating machines. External sensors can also be coupled on the sheath of power cables and on their accessories.

Several studies have been conducted to analyze the benefits of the UHF technique with invasive and external sensors, especially in the resonant metallic chambers of GIS and in power transformers [4,24].

Although wire antennas (dipoles and monopoles), aperture antennas (horn type) and array antennas (log-periodic) are used for PD measurements [25,26,27], the most common UHF sensors for this application are microstrip antennas based on patch type couplers; these consist mainly on a disc or a printed planar resonant structure [28,29,30,31]. This study is focused in the implementation of patch antennas for PD measurements.

**Figure 4:**
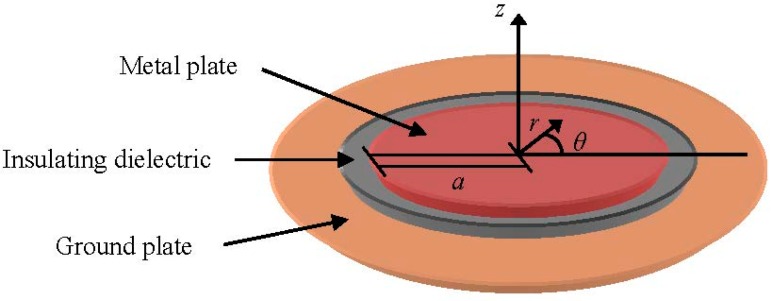
Resonant disc structure for UHF patch sensors.

A patch coupler presents a high immunity to the noise environment because it has a high directivity and measures only EM-wave propagating in one direction. This is an advantage when PD measurements are performed with non-invasive sensors. Patch sensors for measurements in the UHF range consists of a conducting plate above a ground plane, an example of this configuration is shown in Figure 4*.* For the disc resonant structure presented, Stratton’s treatment for the TM wave is applied to the co-ordinate system indicated [32]. Provided that the disc is close to the ground plane in comparison with its radius, two dimensional resonances are considered and the angular resonant frequencies ω*_nm_* are given by:

(4) $${\text{ω}}_{nm}=\frac{{\text{χ}}_{nm}}{a\cdot \sqrt{\text{μ}\cdot \text{ε}}}$$

where *ɑ* is the metal plate radius, μ the permeability of the dielectric, ε the permittivity and χ_nm_ the *m*th nonzero root of *J_n_'*(*x*) = *0*, being *J_n_'(x)* the derivative with respect to *x* of the Bessel function *J_n_*(*x*) of the first kind of order n [29,32,33].

The electric field component E_z_ under the disc at resonance can be expressed with the polar coordinates r and θ:

(5) $$E_{z}=k\cdot \sin\left({\text{ω}}_{nm}\cdot t\right)\cdot J_{n}\cdot \left(\frac{r}{a}\cdot {\text{χ}}_{nm}\right)\cdot \cos\left(n\text{θ}\right)$$

where k is a constant.

For design considerations the resonant frequencies are calculated using Equation (4) and the electric field amplitude E_z_ under the disc for the resonant modes is determined by Equation (5).

Although patch type antennas are resonant in nature and are mainly used in telecommunication applications matched to a particular resonant frequency with a high gain, they can also be designed to match a larger frequency range by increasing the bandwidth of resonance frequencies to be used for PD measurements in a UHF range. Moreover, even though the sensor is not well matched for bandwidths out of the resonance frequencies, PD signals can be also measured with a sufficient gain in these frequency bands. A detailed explanation for the construction and characterization aspects of resonance disc UHF antennas is presented in [29,34]. Furthermore, improvements in the design of this type of UHF sensors to reduce the effect of the coupler on the detected PD spectrum have been presented in the studies developed by [29,30]. Other designs of patch type sensors for PD measurements have been implemented with a different geometrical shape of the patch conductor [21], or with a different configuration of the resonant structure [35].

As was mentioned before PD pulses are very fast, with rise times even below 1 nanosecond, so at their origin they radiate electromagnetic waves with spectral content that can extends to frequencies of 1 GHz or higher. When the discharges are generated inside a GIS compartment or are confined in the enclosure of a power transformer, an electrical resonance is produced that causes the pulses to have a duration of up to 1 µs. Due to the characteristics of the insulating medium that these elements present, the inherent losses of the pulses are very low, for example, in the case of a GIS in the presence of barriers and discontinuities the attenuation is 2 dB/m. However, when the insulation medium is solid, as in power cables, this behaves as a low pass filter and the UHF components of the pulses are significantly attenuated with the distance from the focus [36]. This characteristic is useful to be selective in the location of PD sources when UHF measurements are performed in cables and accessories; if PD pulses are detected in the UHF range the sensor is close to the origin of the defect.

**Figure 5:**
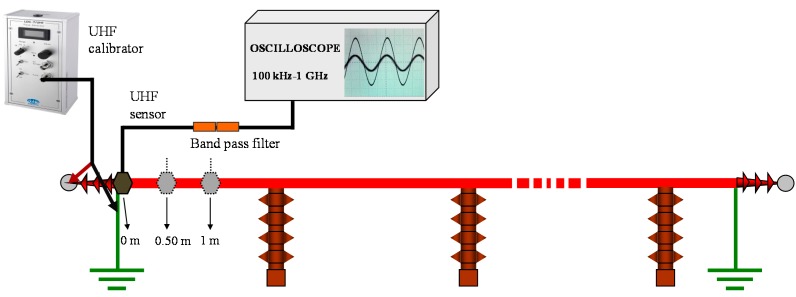
Experimental setup for the evaluation of the UHF frequency components of a PD pulse in a power cable.

An experimental measurement was performed in order to characterize the attenuation of the UHF frequency components of PD pulses propagating in power cables. The experimental setup consisted of a 12/20 kV XLPE cable of 500 m length, see Figure 5. A UHF calibrator (ref. LDIC LDC-5/UHF) was used to inject pulses in the power cable through one of its terminals. The pulses were measured every 0.5 m by means of a UHF non-invasive sensor with a bandwidth from 20 to 800 MHz and a digital oscilloscope of 1 GHz bandwidth and 5 GS/s of sample rate. The measuring frequency range was delimited from 300 MHz to 800 MHz using a band pass filter at the output of the sensor.

**Figure 6:**
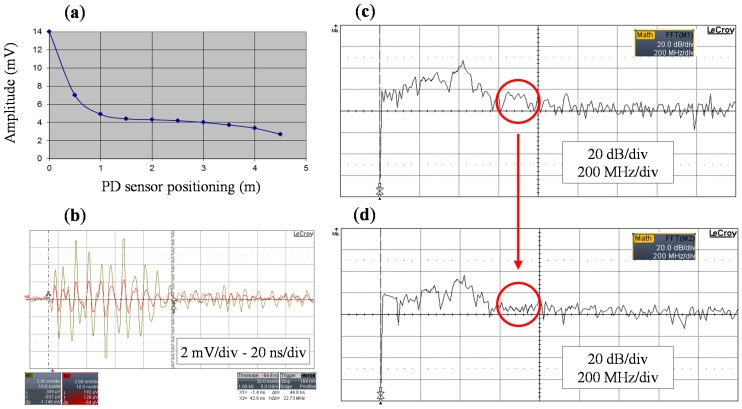
(**a**) PD pulse attenuation with the distance; (**b**) Superposition of a pulse measured at the beginning of the cable and at 3.5 m; (**c**) Frequency spectrum of the pulse measured at 0 m; (**d**) Frequency spectrum of the pulse measured at 3.5 m.

The spatial selectivity for PD location is characterized by a decreasing exponential curve as shown in Figure 6a. In the first 2 m the pulse magnitude is attenuated by 67%. At a distance of 5 m no pulse can be detected with the acquisition system. Figure 6b shows the superposition of a pulse measured at 0 m and at 3.5 m (yellow and red signals respectively). It can be observed that the amplitude decreases and that the shape of the signal changes with the distance covered. Comparing the frequency spectrum of both signals, the amplitude values obtained for the frequency range from 600 to 800 MHz when the sensor is positioned at the beginning of the cable are negligible when the sensor is at 3.5 m, see Figure 6c,d. This characterization confirms that the location of defects in power cables can be performed in a very selective way.

The following advantages are obtained when PD measurements are performed with UHF sensors.

- High immunity to electric noise, interferences and corona discharges in air, since the frequency spectrum of these signals in the UHF range is very low and in some emplacements negligible.
- High sensitivity in PD detection inside shielded GIS compartments, metal enclosed switchgears and transformer tanks, due to the inner electrical resonance, very low inherent losses and low noise levels.
- Possibility of PD source location. In the case of GIS and transformers the location is performed using several sensors and analyzing the arrival times of the pulses (time-of-flight measurements).
- Accurate defect locations are achieved in cables and accessories due to the selectivity in the distance that this technique presents.
- Possibility to discriminate between internal and external defects to the HV equipment under consideration.

**Figure 7:**
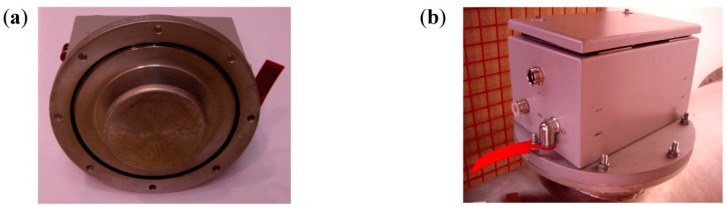
(**a**) Metal plate of the invasive UHF sensor; (**b**) External view of the sensor installed in a GIS compartment.

In the study presented two types of commercial UHF patch sensors were used to perform the measurements together with the UHF-HF converter developed. The first one is an invasive disc coupler specially design for GIS compartments and metal enclosed switchgears, with a bandwidth form 0.1 to 1.5 GHz and a load impedance recommended of 50 Ω (ref. Siemens 926 98850 174 B). Figure 7 shows the metal plate of the sensor and a view of its installation in a GIS compartment.

**Figure 8:**
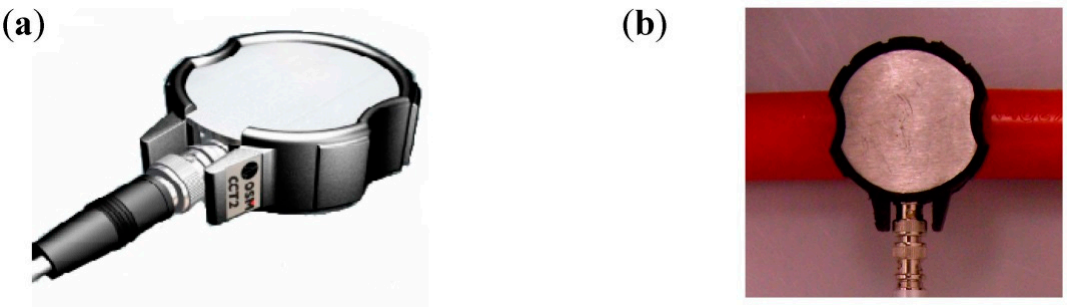
(**a**) UHF non-invasive patch coupler; (**b**) Sensor installed on the surface of a power cable.

The other type of patch sensor is a non-invasive antenna (ref. IPEC OSM CCT2) designed to be coupled in the exposed barrier edges of the metal cladding of GIS, switchgear cabinets or power transformers and on the surface of power cables and their accessories, see Figure 8. It has a bandwidth of 20–800 MHz and its recommended load impedance is 50 Ω.

For PD measurements in UHF, where the noise level is very low, the use of an amplifier connected to the output of the sensors is recommended, since when low amplitude pulses are captured, this element helps to achieve more sensitivity when they are recorded by the measuring equipment. Furthermore, when the pulses present an amplitude slightly above the noise level, the use of an amplifier helps to differentiate in more detail a possible PRPD pattern. In this study, the PD measurements with UHF sensors have been performed with an amplifier of 20 dB. This amplifier has a bandwidth from 100 kHz to 1 GHz and an input impedance of 50 Ω.

The measurements with the UHF non-invasive sensor have been performed delimiting its bandwidth in a defined frequency range starting from 300 MHz. By doing this, a greater immunity to noise and interference signals of low and high frequency is obtained, being the signal to noise ratio (SNR) higher. Furthermore, as was explained before, PD measurements in cable accessories from 300 MHz allow more selectivity in the location of defects. Considering the importance of these two advantages, a high pass filter of 300 MHz was connected at the output of the non-invasive UHF sensor. Besides, in order to avoid radiofrequency disturbances mainly generated by telecommunication applications it is recommendable to implement also a low pass filter of 800 MHz.

**Figure 9:**
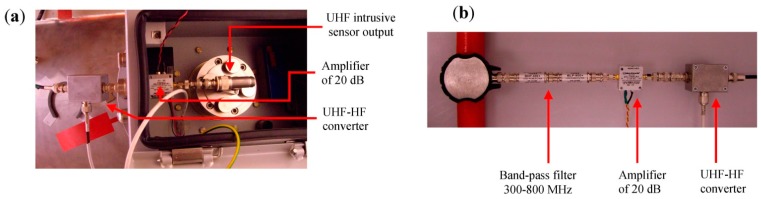
(**a**) Outside view of the UHF invasive sensor with amplifier and UHF-HF converter; (**b**) Non-invasive sensor with band-pass filter, amplifier and converter.

Figure 9a shows the configuration of the UHF invasive sensor with the amplifier and the UHF-HF converter connected to its output. The final configuration of the non-invasive sensor coupled on a cable with the band-pass filter (300–800 MHz), the amplifier and the converter is shown in Figure 9b.

### 2.3. UHF-HF Converter

Processing PD signals measured in the UHF frequency range requires oscilloscopes with a bandwidth of at least 1 GHz and a 5 GS/s sampling rate. These oscilloscopes are very expensive and are not provided with a specific software designed to record and analyze the raw data acquired with UHF sensors.

Commercial PD instruments designed to measure in the HF range with a sampling rate around 100 MS/s, can be used to record and process the measured UHF signals after they are transformed into HF signals by means of a frequency converter; these instruments are less expensive than the oscilloscope specified to measure in UHF. The UHF-HF converter developed in this study enables to take advantages of capturing the pulses in UHF and in turn record and analyze the signals with a measuring instrument designed to perform PD measurements with HF sensors.

**Figure 10:**
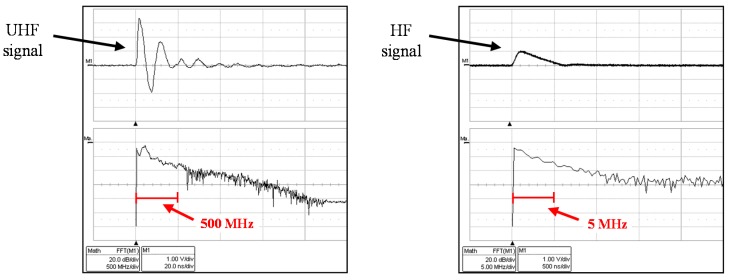
Frequency analysis of an original UHF pulse and the transformed signal.

The design of the converter is based on an integrator model that presents a delay in the output signal of less than 40 ns. The converter detects the peak of the UHF input signals and applies a level shift to convert the signals to continuous levels. Furthermore, it is integrated with an internal amplifier which gain can be adjusted. Its bandwidth is from 0.1 to 3 GHz. Figure 10 shows a pulse measured in UHF and the same signal converted in a HF pulse. It can be observed that the original signal has significant values of amplitude in the frequency spectrum up to 500 MHz, while the output signal of the converter presents significant values up to 5 MHz.

## 3. Measuring Instrument and Signal Processing Tools

### 3.1. Measuring Instrument

**Figure 11:**
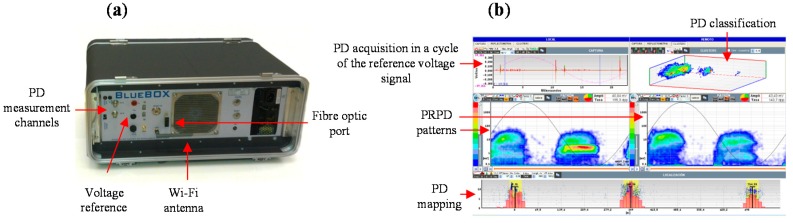
(**a**) Measuring instrument; (**b**) Display of an acquisition in a PD test.

A measuring device was designed to perform PD measurements in on-line conditions. It is equipped with an acquisition board of 14 bits of vertical resolution, a bandwidth of 50 MHz and a sampling frequency of 100 MS/s. The signal processing tools described below were developed and integrated in the processing and analysis stages of the PD instrument in order to improve the ability to perform accurate diagnosis. The equipment is controlled by a computer connected via fiber optic cable or through a wireless connection. The front view of a measuring unit and the display screen for PD acquisition and post processing is shown in Figure 11. To be measured by this measuring instrument, pulses captured with UHF sensors must be converted to HF pulses with frequency content at most 50 MHz. This conversion is performed with the UHF-HF converter described in previous section.

### 3.2. Signal Processing Tools

The effectiveness of the new developments and applications of DP processing tools has increased the use of PD measurements for maintenance purposes. By implementing certain processing tools, an accurate assessment of the HV elements insulation condition can be achieved. Three processing tools have been designed and implemented in the measuring instrument developed: noise filtering, automatic PD location and PD sources classification [37].

#### 3.2.1. Noise Filtering Tool

**Figure 12:**
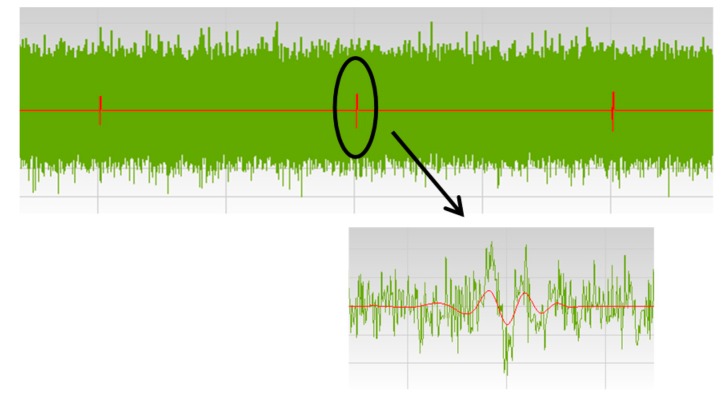
Result of the filtering tool. Discrimination of pulse-shaped signals overlaid with background noise.

A filtering tool based on the wavelet transform (WT) makes it possible to discriminate pulse signals from continuous background noise. Much effort has been focused on de-noising and detecting transient signals implementing wavelets algorithms [38,39]. The filtering tool developed is based in the use of the WT, together with a statistical analysis that evaluates the standard deviation of the background noise level to discriminate the existence of PD activity. Applying the implemented wavelet filter, pulse-shaped signals with amplitudes even below the background noise level can be detected, see Figure 12.

The signals presenting a transient behavior (PD and pulse-type interferences) are filtered and selected for further processing. The most significant difference with traditional PD instruments is that for the pulse selection is not necessary to set any noise threshold level; all those pulses filtered by signal processing are recorded.

After the filtering process not all the selected signals correspond to PD pulses as it is not possible to discriminate pulse-type disturbances presenting a transient behavior similar to PD. Nevertheless, applying the classification by location tool and the sources classification tool enables distinguishing clusters associated to PRPD patterns corresponding with this type of noise sources.

#### 3.2.2. Classification by Location Tool

**Figure 13:**
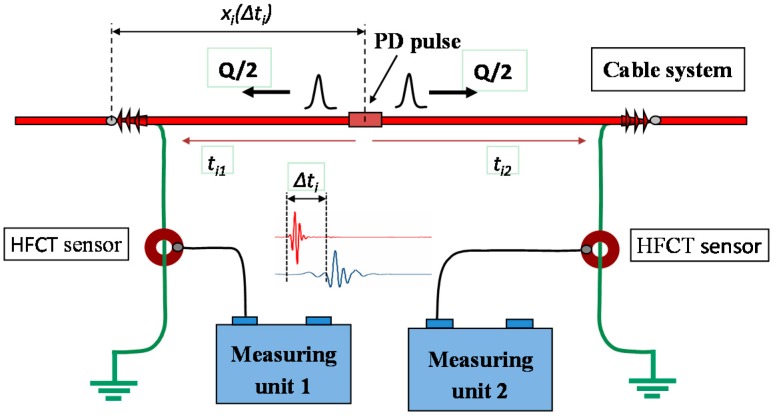
Time delay between the arrival times to the measuring units.

When a pulse source is present in a HV equipment the signals generated propagate through the grid in different directions according to the impedance characteristics of the medium. As an example, in the case of a cable system two pulses travel towards opposite directions along the cable length from the source site. Each pulse arrives in a certain instant *t_i_* to a PD sensor connected to a measuring unit, see Figure 13.

The synchronization of different measuring units for PD location can be accomplished in different ways: by sending a synchronizing signal through a fiber optic cable, by the time reference of the coordinated universal time (CUT) from the pulse per second signal (PPS) of a global positioning system receiver (GPS), or by a reference signal sent through the installation under test from a measuring unit to the others. In this study the synchronization by GPS has been used. The PPS is generated by a GPS module that achieves an accuracy within 15 ns (1σ).

A pulse recorded in a measuring unit is correlated with another pulse recorded by other unit only if the time delay between the arrival times of both pulses Δ*t_i_* is less than the propagation time between sensors *t_w_* defined in Equation (6):

(6) $$t_{w}=L/V$$

being *L* the distance between sensors and *V* the propagation speed of the signals.

By determining the time delay Δ*t_i_* for the correlated pulses captured by each sensor, the measuring system is able to establish automatically the location of the different pulse sources. On the basis of the knowledge of the propagation time *t_w_* and the cable length *L*, the location of a pulse source *x_i_(*Δ*t_i_)* for the correlated pulses is established by the following expression:

(7) $$x_{i}\left(\text{Δ}t_{i}\right)=\frac{L}{2}\cdot \left[1-\left(\frac{\text{Δ}t_{i}}{t_{w}}\right)\right]$$

where:

(8) $$\text{Δ}t_{i}=t_{i2}-t_{i1}$$

**Figure 14:**
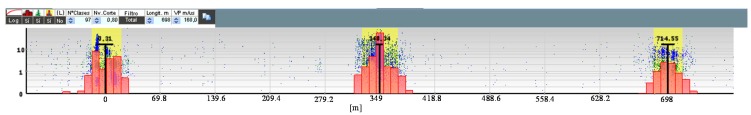
Example of pulse classification by location. PD sources located in different sites of a cable system.

All correlated pulses are positioned in a PD mapping diagram and the different locations of pulse sources are identified (see Figure 14).

#### 3.2.3. PD clustering Tool

**Figure 15:**
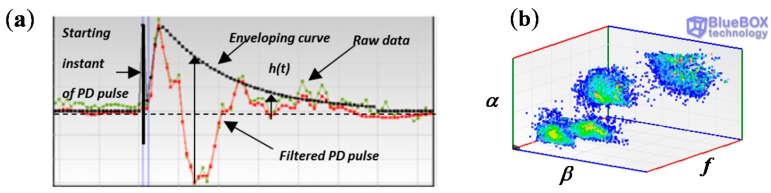
(**a**) Enveloping function for a filtered PD pulse; (**b**) 3D diagram for the classification of pulse sources by clusters.

When more than one PD source is present in a HV installation and especially if their emplacement is in the same position it is necessary to differentiate and to identify them. In this processing tool, a damped oscillating wave *f_i_(t)* is associated to each filtered pulse, that is modeled by means of a sine function *g_i_(t)* and modulated by an enveloping function *h_i_(t)* that fits the local maxima of the absolute values of the signal as shown in Figure 15a:

(9) $$f_{i}\left(t\right)=g_{i}\left(t\right)\cdot h_{i}\left(t\right)$$

(10) $$g_{i}\left(t\right)=sin\left(w_{i}\cdot t-{\text{φ}}_{i}\right)$$

(11) $$h_{i}\left(t\right)=\frac{A_{i}}{e^{{\text{α}}_{i}\left(t\right)}+e^{-{\text{β}}_{i}\left(t\right)}}$$

The parameter *w_i_* = *2*π*f_i_* for *g_i_(t)* is estimated using the Fourier transform of the filtered signal and corresponds with the maximum amplitude value of the spectrum. The shape of the enveloping function *h_i_*(*t*) is mainly described by the damping coefficients of the exponential terms α and β, which are related with the steepness in the increasing and decreasing intervals, respectively; the parameter *A_i_* characterizes the amplitude of the envelope. By adjusting the values of α, β and *A_i_* it is possible to obtain a suitable enveloping function to modulate the sinusoidal waveform. The criterion to select the best fitting for the function *h_i_*(*t*) is the least squares. The parameters of the modeled pulses *f_i_,* α*_i_* and β*_i_* characterize their waveform providing useful information for the classification by clusters of the pulses originated in different sources of the HV installation. Implementing the three dimensional diagram shown in Figure 15b using these waveform parameters, different pulse sources can be distinguished by selecting the formed clusters.

## 4. HF and UHF PD Measurement

In order to analyze the advantages of the complementary measurement with HF and UHF sensors applying suitable processing and analysis tools, four types of insulation defects were generated and measured simultaneously in a laboratory experimental setup. The PD sources implemented were: two internal defects in XLPE, corona in air and corona in SF_6_ inside the metal cladding of a GIS compartment.

### 4.1. Generation of PD Sources

Internal discharges (voids inside solid dielectrics) are considered the most harmful for dielectric elements and lead to insulation failures. Corona discharges pose no risk if they are generated in air, however, if they are produced close to silicone insulators or in SF_6_, the consequences also lead to insulation faults.

**Figure 16:**
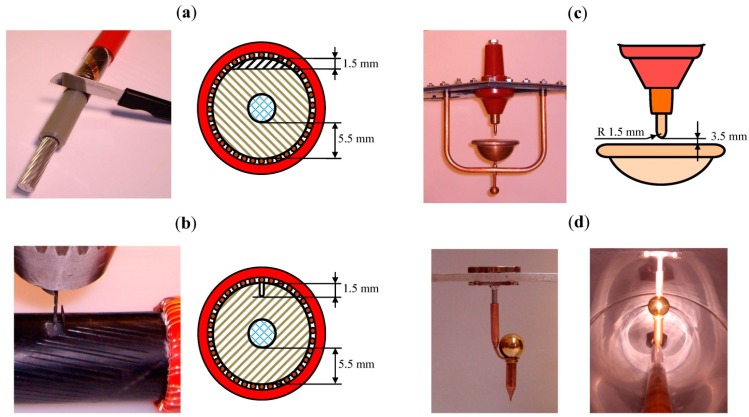
PD sources. (**a**) Transversal cut in the main insulation of a cable termination. (**b**) Internal void inside the main insulation of a cable; (**c**) Point-plane corona in air; (**d**) Corona in a GIS chamber.

*Internal defect type 1*. A cavity type defect was caused in a cable termination at the end of the semiconducting external layer. The cavity was done by a transversal cut of 1.5 mm depth in the main insulation (XLPE), as shown in Figure 16a. This type of defect can be caused due to an incorrect handling of the splicing tools at the edges of semiconducting shield cut-backs, in accessories assembling processes [20].

*Internal defect type 2*. Another cavity type defect was caused in a cable termination making a void in the main insulation (XLPE). A hole of 1.5 mm depth was drilled using a 1 mm drill bit removing previously a part of the cable semiconducting external layer, as shown in Figure 16b. The semiconducting layer was fixed again to the main insulator and the cuts were reconstructed using a semiconducting varnish (ref. Raychem EPPA 220). This type of defect occurs due to failures in the cable extrusion process that causes a void in the main insulation, or when a void remains inside the insulation elements after the assembly process of the cable accessories.

*Corona in air (tip as HV electrode).* Corona discharges were caused by means of a point-plane configuration assembled in a holding device that allows the adjustment of the gap between the point and the plane electrodes (see Figure 16c).

**Figure 17:**
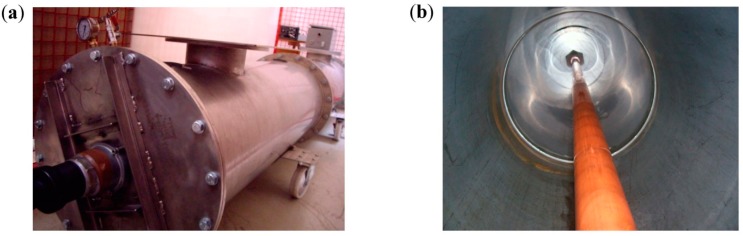
(**a**) Outside view of the GIS compartment; (**b**) Interior view.

*Corona in a GIS compartment (tip as ground electrode).* To simulate a corona defect in SF_6_, a 1 mm radius tip was fixed in the enclosure of a GIS compartment that was manufactured with a stainless steel metal cladding of 3 m length. This chamber has a thickness of 3 mm and an external diameter of 500 mm. A conductor of 54 mm diameter was assembled inside the compartment, see Figure 17b. The internal conductor was supported by two epoxy resin cone spacers. The metal cladding is provided with two inspection windows available for the assembling of invasive PD sensors as shown in Figure 17a.

The mechanical and electrical connection of the tip with the metal cladding was performed using neodymium magnets, which provides a reliable joining and prevents the machining of the structure, see Figure 16d. The gap between the tip and the internal conductor can be adjusted with a screw. For a gas pressure inside the compartment of 4 bars and applying a test voltage of 20 kV, the distance between the tip and the internal conductor was adjusted in 3 mm. Figure 16d shows the tip assembled inside the GIS chamber. In practice, corona discharges in SF_6_ can be caused by protrusions due to errors in the installation process, to the friction between moving parts, to burrs or to material degradations caused by switching operations during the service life of the GIS [40].

### 4.2. Experimental Setup

**Figure 18:**
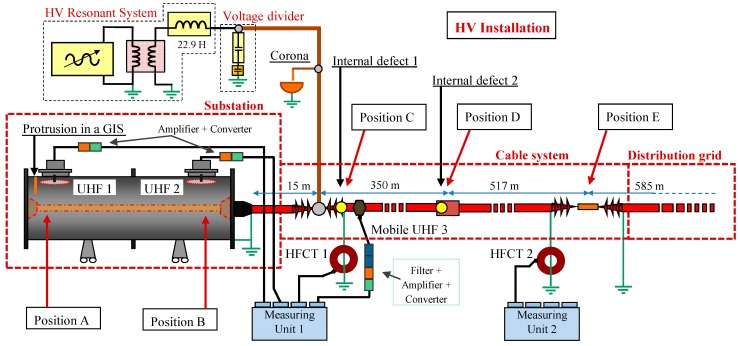
Experimental setup for the measurement of insulation defects with HF and UHF sensors.

An experimental arrangement implemented in a HV laboratory was configured in order to simulate a representative HV installation and to perform significant DP measurements with the proposed sensors. This installation was composed by the following subsystems, see Figure 18.

(a) *Substation.* Formed by the GIS compartment described above and a 12/20 kV XLPE cable of 15 m length and a 240 mm^2^ aluminum conductor. This cable interconnects by a plug-in terminal (position B) and a cable junction (position C) the GIS compartment with the cable system.
(b) *Cable system.* A 12/20 kV XLPE cable system with a 240 mm^2^ aluminum conductor was configured with a total length of 867 m. The line was composed by two joined cable coils with lengths of 350 and 517 m. The connection between them (position D) was made with a splice.
(c) *Distribution grid.* To simulate the continuity of the distribution grid a 12/20 kV XLPE cable system with a total length of 585 m and a 240 mm^2^ aluminum conductor was connected to the cable system. The connection (position E) was made simulating a junction of the switchgear cabinets of a distribution transformer substation.

A resonant generator of 36 kV with a 22.9 H reactor was used to apply a test voltage of 51 Hz and 20 kV. This voltage level was above the inception voltage of each defect and provided stable measurements. The HV generator was connected between the cable system and the connecting cable with the GIS chamber (position C). In this point the synchronization reference signal for the PD measurements was acquired by a capacitive divider.

The four insulation defects described above were measured simultaneously in the HV installation arranged simulating an on-line test. These defects and their position in the test object are specified in Table 1.

**Table 1:** Type of defect and location.

Insulation Defect	Location
1. Corona in a GIS compartment	A (inside the GIS chamber)
2. Internal defect type 1 in a cable termination	C (in the power cable)
3. Corona in air	C (in the power cable)
4. Internal defect type 2 in one of the cable terminations of a splice	D (in the power cable)

### 4.3. Measuring Procedure

In order to be effective enough in the detection and identification of defects, in a first step the measurements were performed with two HFCT sensors for the cable system and with two UHF invasive sensors for the GIS compartment. The measurements with these sensors placed in strategic positions, performed with the measuring instrument described in Section 3.1, present a good technical and economic solution for the complete evaluation of the HV installation.

Due to the attenuation and dispersion effects of PD signals in power cables until they are captured by the HFCT sensors, the maximum distance between sensors recommended is around 1 km. This distance can be increased to 2 km at the expense of a loss of sensitivity that is compensated by the noise filtering technique applied. In order to gain a general overview of the entire setup proposed, the HFCT sensors were installed in the ground connections of the cable system in positions C and E (see Figure 18), being the distance between them 867 m.

By coupling invasive UHF sensors in the GIS compartment, defects in its interior can be detected and located with high sensitivity. To provide a proper coverage of the substation in terms of sensitivity when PD measurements are performed in GIS, it is recommended to couple a UHF sensor approximately every 15–20 m. Due to the dimensions of the GIS chamber implemented in the experimental setup, the two invasive UHF sensors were placed inside the compartment with a distance between them of only 2 m.

In a second step, if any insulation defect is detected with the HFCT sensors, a mobile non-invasive UHF sensor can be coupled in different sites of the HV installation to determine the exact position of the PD source. A summary of the sensors and their position in the test object is presented in Table 2.

**Table 2:** Type of sensors and location in the test setup.

Invasive UHF Sensors	Position
UHF 1	A
UHF 2	B
**Non-invasive HFCT Sensors**	**Position**
HFCT 1	C
HFCT 2	E
**Non-invasive UHF Sensor**	**Position**
Mobile UHF 3	In different emplacements along the HV installation to determine the exact PD source location

The PD measurements were performed with two measuring instruments of four acquisition channels. Table 3 indicates the connection of the sensors to the measuring units.

**Table 3:** Measuring instrument and channel assigned to each sensor.

Measuring Instrument	Channel	Sensor
**Measuring Unit 1**	Channel 1	Invasive UHF 1 (position A)
	Channel 2	Invasive UHF 2 (position B)
	Channel 3	HFCT 1 (position C)
	Channel 4	Mobile non-invasive UHF 3
**Measuring Unit 2**	Channel 1	HFCT 2 (position E)
	Rest of channels	Free for more sensors if required

### 4.4. Experimental Results and Analysis

The performance of the described sensors and processing tools is validated by the measurement of the PD sources in the presence of noise interferences. A significant amount of pulses were processed in order to obtain representative patterns for the individual sources. In practice, a thorough PD analysis is required, since up to four defects are present in the HV installation, with the added difficulty of the emplacement of two of them in the same site (position C).

**Figure 19:**
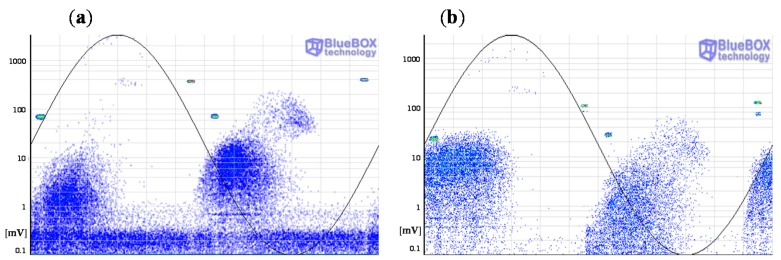
PRPD patterns obtained with the HFCT sensors. (**a**) HFCT 1 in position C; (**b**) HFCT 2 in position E.

Firstly the measurements performed with the HFCT sensors are analyzed. The PRPD patterns for the complete acquisition obtained with both sensors after the application of the wavelet noise filter are shown in Figure 19. As the individual patterns generated in each source are mixed, the insulation condition of the HV system can not be determined in a first assessment.

**Figure 20:**
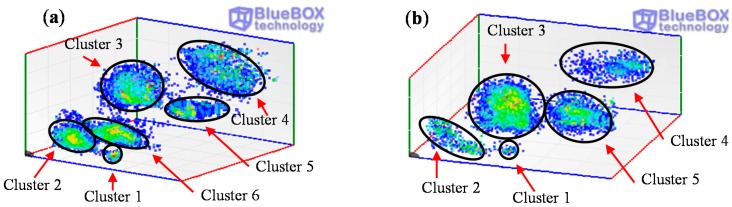
Result of the PD classification tool for the acquisitions with HFCT sensors 1 (**a**) and 2 (**b**).

A further analysis is required for an accurate identification of the defects. In a first step the pulse-type noise sources and interferences measured in the HV installation are removed. Pulse-shaped electric noise can be differentiated from PD pulses with the PD classification tool by pulse waveform. The results of this processing tool are shown in Figure 20. For the measurement performed with the sensors HFCT 1 and HFCT 2, 6 and 5 clusters have been differentiated respectively. Analyzing the PRPD patterns for each group of pulses it has been possible to correlate all the clusters obtained with each sensor except the cluster 6 corresponding to the sensor HFCT 1.

**Figure 21:**
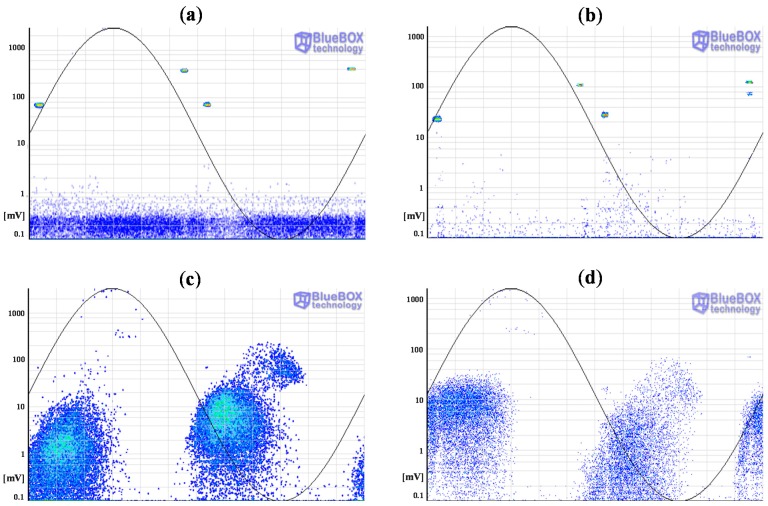
PRPD patterns of the noise pulses measured with the HFCT sensor 1 (**a**) and HFCT sensor 2 (**b**) corresponding to clusters 1 and 2. PRPD patterns of the PD pulses measured with the HFCT sensor 1 (**c**) and 2 (**d**).

By selecting the pulses corresponding to cluster 1, the electronic noise generated by the power electronic (IGBTs) of the resonant system used to apply high voltage is identified. These interferences are synchronized with the test voltage reference signal. Furthermore, a significant amount of random pulse-shaped signals conducted by the earth network of the installation were also identified by selecting the pulses of cluster 2. The PRPD patterns corresponding to clusters 1 and 2 are shown in Figure 21a,b. Once these noise signals are removed, the remaining pulses considered PD, are shown in Figure 21c,d.

**Figure 22:**
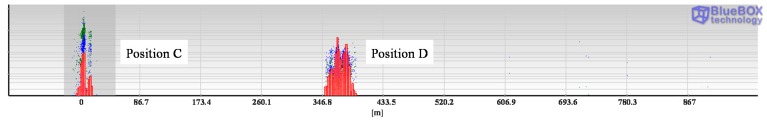
PD classification by location. PD sources located in positions C and D of the cable system.

In a second step, the classification by location tool is applied to the PD pulses and the PD mapping shown in Figure 22 is obtained. Analyzing the time delay Δ*t_i_* for correlated pulses, two different emplacements of PD sources were detected (positions C and D in Figure 18 and Figure 22). These sites correspond with the emplacement of the sensor HFCT 1 and with the joint in the cable system respectively.

**Figure 23:**
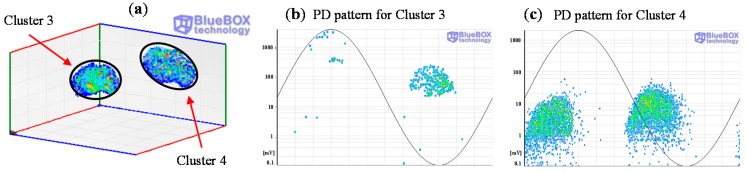
(**a**) Classification of PD pulses positioned in C, (**b**) and (**c**) PRPD patterns for the sources positioned in C.

In a third step, the classification tool by pulse waveform is applied to those pulses corresponding with a specific location. In position C two clusters have been identified for the measurements performed with sensor 1, see Figure 23. These two groups of pulses correspond with clusters 3 and 4 in Figure 20a. The individual PRPD patterns are obtained by selecting the formed clusters, see Figure 23b,c.

The pattern displayed for cluster 3 is characteristic of a corona defect (tip as HV electrode). PD pulses appear on the crests of the reference voltage signal. There are stable amplitude values within a certain range in both half-periods and there are less pulses and with higher amplitude in the positive half-cycle.

The pattern corresponding to cluster 4 is characteristic of an internal defect in a solid dielectric. PD pulses occur slightly before the zero crossings and in the increasing intervals of the voltage signal. There is certain symmetry and a similar repetition rate when comparing the patterns of both half-periods.

**Figure 24:**
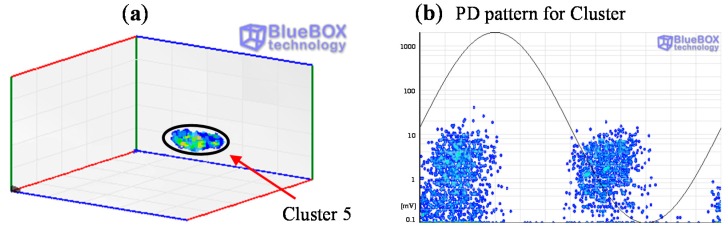
(**a**) Classification of PD pulses positioned in D; (**b**) PRPD pattern for the source positioned in D.

In position D only the group of pulses corresponding to cluster 5 was identified (see Figure 20a and Figure 24a). The pattern obtained is also characteristic of an internal defect, see Figure 24b. The patterns shown for positions C and D were obtained for the signals acquired by the sensor HFCT 1.

**Figure 25:**
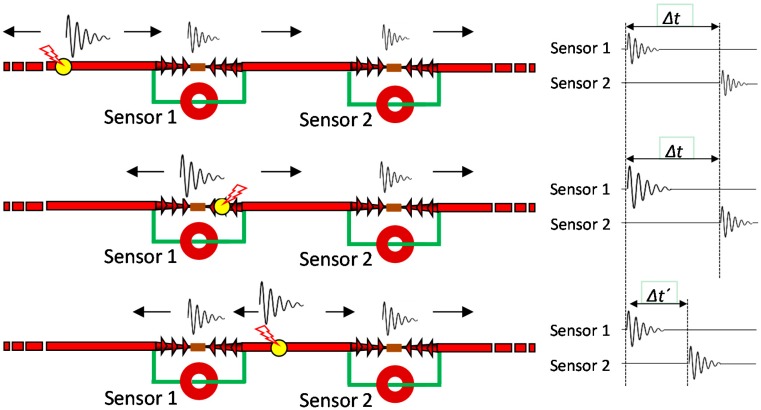
(**a**) Defect before the section of cable between sensors; (**b**) Defect located in the same emplacement as one of the sensors; (**c**) Defect inside the section of cable between sensors.

In order to corroborate the location of the defects in the HV installation, it must be considered that:

- When the pulses are positioned by the location tool in the same site where a HFCT sensor is coupled the location of the defect can not be totally assured, *i.e.*, the source can be in that position or in a previous one. This is because in both cases the delay in the arrival time of the pulses to the sensors is the same, see Figure 25a,b, so the pulses are always positioned in the emplacement where the HFCT sensor is placed.
- Only when the defect is in an intermediate point between the sensors it can be assured that the positioning of the focus is correct; a certain time delay corresponds only to one emplacement of the PD source, see Figure 25c.

According with this and considering the internal defects indicated in the PD mapping it can be assured only the correct emplacement for the defect of position D (positioned between the sensors).

The emplacement of the corona defect in position C can also be verified, as it is only in this point where the high voltage conductor of the cable system is exposed to air.

**Figure 26:**
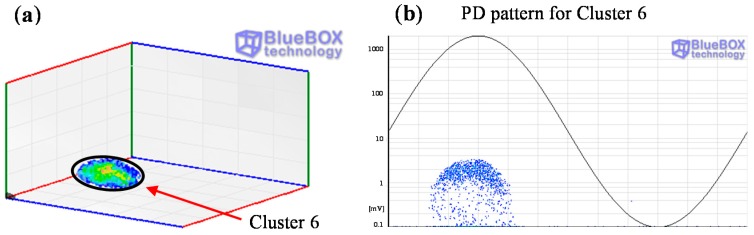
(**a**) Cluster 6 detected with the sensor HFCT 1; (**b**) PRPD pattern corresponding to cluster 6.

All clusters obtained with the classification tool by pulse waveform have been analyzed except cluster 6, see Figure 20a and Figure 26a. The PD source corresponding with this cluster was detected with the sensor HFCT 1 but not with the HFCT 2 due to the attenuation effect of the pulses, so the location of this source was not possible. Analyzing the characteristics of the pattern it seems that it is related with a corona defect (tip as ground electrode), probably located in the GIS compartment.

Once analyzed the results obtained with the measurements performed applying the HFCT sensors the following statements can be made.

- In position C two types of sources were identified: corona in air and an internal defect.
- The location of the corona defect in position C was ratified. However, the emplacement of the internal defect could not be confirmed.
- In position D an internal defect in a joint was detected and located.
- A corona type PD source related with a tip referenced to ground (cluster 6) was detected with the sensor HFCT 1. The location of this source could not be determined.

In order to complement the previous diagnosis, the measurements performed with the invasive UHF sensors were analyzed. Furthermore, to confirm the location of the internal defect in position C, the mobile non-invasive sensor UHF 3 was coupled in each cable termination of this emplacement.

**Figure 27:**
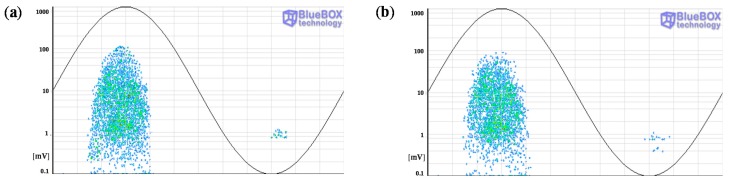
PRPD patterns obtained with the UHF sensors. (**a**) Sensor UHF 1 in position A; (**b**) Sensor UHF 2 in position B.

The PRPD patterns for the acquisition with the invasive couplers located in positions A and B of the GIS compartment are shown in Figure 27a,b. Due to the selective approach of the measurements in UHF only a single pattern has been obtained for each sensor. Moreover, it can be noticed the immunity that this technique presents to background noise interferences.

The PRPD pattern detected with the invasive UHF sensors in the GIS compartment is characteristic of a corona defect in SF_6_ with a tip referenced to ground. PD pulses appear on the crests of the reference voltage signal and there are more pulses in the positive half-cycle.

As the captures were performed with invasive UHF sensors, it can be assured that the pulses were generated inside the compartment; UHF sensors are insensitive to the low frequency spectrum of the pulses generated in the cable system that propagate to the GIS. Furthermore, this PRPD pattern has the same shape as the one measured with the sensor HFCT 1 corresponding with cluster 6, so it can be concluded that the non-localized corona defect measured with this sensor is the one generated in the GIS chamber.

**Figure 28:**
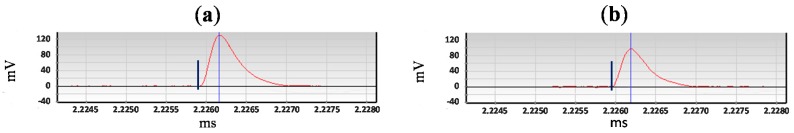
(**a**) PD pulse measured with the sensor UHF 1 and converted into a HF pulse; (**b**) PD pulse measured with the sensor UHF 2 and converted into a HF pulse.

Due to the high propagation speed of PD signals inside the GIS compartment (0.28 m/ns) and the short distance between sensors (2 m), in this case the localization tool can not be applied, as the sampling period of the measuring instrument is 1 sample every 10ns. An approximate location of the defect is possible comparing the amplitude of the integrated pulse of a PD measured with both sensors; the coupler with the higher signal level is closer to the PD source. The higher amplitude obtained for the pulse measured with the sensor UHF 1 evidences that the defect is closer to the position A, see Figure 28. An accurate location can be obtained by analyzing the travelling time of the UHF signals measured with a dual-channel oscilloscope of at least 1 GHz of bandwidth and 5 GS/s of sampling rate.

**Figure 29:**
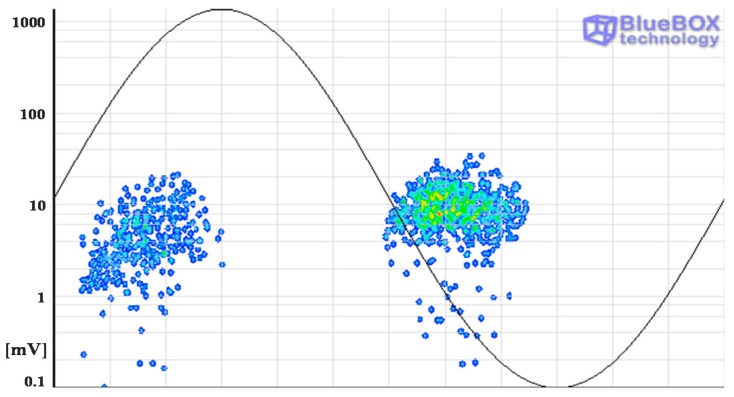
PRPD pattern obtained with the mobile non-invasive UHF sensor coupled in the right termination of position C.

Finally, the PRPD pattern shown in Figure 29 was obtained with the mobile sensor UHF 3 coupled in the right cable termination of position C. No PD signals were detected in the left termination. This pattern is characteristic of an internal defect and is very similar to the one obtained with the sensor HFCT 1, see Figure 23c. The detection with the sensor UHF 3 of this internal pattern confirms that the PD source is in this cable termination.

The measurements performed with the UHF sensors made it possible to identify and locate correctly the corona defect of the GIS compartment and also to confirm the emplacement of the internal defect in the right cable termination of position C. The complementary measurements with both techniques enabled the identification and location of all the insulation defects generated in the HV installation.

## 5. Conclusions

In the study presented theoretical and practical aspects about the application of HF and UHF sensors for PD measurements have been considered and analyzed. The use of HFCT sensors with a bandwidth of 20 MHz is recommended for their practical implementation in on-line PD measurements.

For the measurements performed with UHF sensors the use of a UHF-HF converter enables to take advantages of capturing the PD pulses in the UHF range and to process them in the HF range. Therefore, using only a PD instrument with a bandwidth covering the HF range, the signals measured with HF and UHF sensors can be analyzed. Several insulation defects present simultaneously in a HV installation can be detected and located applying the measuring and analysis procedure described in this paper.

Representative measurements have been performed with the PD instrument developed, in order to show the effectiveness and advantages offered by the complementary measurements with HF and UHF sensors. The information provided measuring in the HF and UHF frequency ranges and the analysis of all the individual PRPD patterns obtained provide the knowledge necessary to make accurate diagnoses.

The measurement and analysis techniques presented, with the combination of the different sensors and processing tools can be employed to accurately assess the insulation condition of HV grid network assets in on-line measurements or in continuous monitoring applications.
